# Emergent Laparotomy Reveals Possible Meckel’s Diverticulum: A Case Report

**DOI:** 10.7759/cureus.71498

**Published:** 2024-10-14

**Authors:** Casey O'Doherty, Audrey Yan, Missy O'Doherty, Denise Gilman

**Affiliations:** 1 Medicine, West Virginia School of Osteopathic Medicine, Lewisburg, USA; 2 Emergency Medicine/Paramedics, Institute of Applied Technology, San Diego, USA; 3 General Surgery, Trinity West Medical Center, Steubenville, USA

**Keywords:** abdominal laparotomy, meckel's diverticulum complications, perforated meckel's diverticulum, small bowel diverticula, small bowel obstruction

## Abstract

Small bowel obstruction (SBO) remains the most common diagnosis encountered by general surgeons, with 70% of cases related to adhesions from previous abdominal surgeries. Less common etiologies include Crohn’s disease, gallstone ileus, and Meckel’s diverticulum (MD). While MD is the most common congenital anomaly of the gastrointestinal tract, it is less frequently considered as a cause in adults. Nonetheless, it remains crucial to consider MD as a potential cause of SBO in adults, especially when evaluating patients with unexplained SBO and recurrent idiopathic abdominal pain, especially in those without a history of abdominal surgery. We present a case of a 74-year-old male presenting with right upper quadrant abdominal pain, constipation, and bilious vomiting, with a presumed diagnosis of SBO and perforation, potentially attributed to MD. This case highlights the differential challenges posed by small bowel diverticula and their complications, underscoring the need for vigilance in recognizing these complications and optimizing clinical management.

## Introduction

Small bowel diverticula are protrusions within the small bowel. Their occurrence is notably infrequent but can give rise to various complications. One of these complications that is cause for significant concern is a small bowel obstruction (SBO). The diverticular structures exert pressure on specific regions of the bowel, resulting in mechanical obstruction within the affected area [1]. This mechanical obstruction can lead to the need for urgent surgery in the presence of ischemia or perforation [2]. The diagnosis can be made with computed tomography (CT) of the abdomen and pelvis without contrast [3]. This case discusses a patient with an undiagnosed small bowel diverticulum which led to obstruction and perforation. After the emergent laparotomy, there was a high clinical suspicion that Meckel’s diverticulum (MD) was the cause of the obstruction.

While MD is most commonly found in children and deemed rare in adults, several reported adult cases in recent years have shown similar findings, and commonly manifest as intestinal obstruction and potential perforation, often uncovered during laparotomy. MD was first described in 1650 by Fabricus Heldanus, and eventually named by Johann Friedrich Meckel in 1809 who discovered its embryonic origin. MD is the most common congenital anomaly of the gastrointestinal tract. Its characteristics mainly adhere to the “rule of twos,” which states that it occurs in 2% of the population, 2 feet proximal to the ileocecal valve, 2 inches in length, and has a male-to-female ratio of 2:1 [4,5]. Originating from the partial degeneration of the omphalomesenteric canal during embryonic development, it constitutes a protrusion of all three layers of the intestinal wall - known as a true diverticulum - within the small intestine and encompasses 97% of all omphalomesenteric duct malformations [5,6].

The most significant challenge for diagnosing MD is pre-op diagnosis. If perforation is present, upright chest and abdomen radiographs will show free air under the diaphragm. Barium studies and CT scans are also not beneficial at detecting diverticula but may reveal gas-filled structures when distension develops. The technetium-99m pertechnetate scan is diagnostic for MD and is commonly used due to its non-invasive nature. While this scan is 95% specific and 80-90% sensitive in children, it is only 9% specific and 62.5% sensitive in adults [5]. These disparities in testing outcomes could pose challenges for accurate diagnoses in adults with MD, even in non-acute settings, and could potentially lead to life-threatening complications if misdiagnosed or missed. While many of these scans are useful in patients presenting with complications associated with MD, none are able to definitively give a pre-surgical diagnosis, especially in an emergent setting.

## Case presentation

A 74-year-old male with no significant past medical or surgical history presented to the emergency department (ED) with a five-day history of right upper quadrant (RUQ) abdominal pain, constipation, decreased appetite, and bilious vomiting. A physical examination revealed a notably distended abdomen, bowel sounds were decreased, and tenderness to palpation in RUQ without peritonitis.

**Figure 1:**
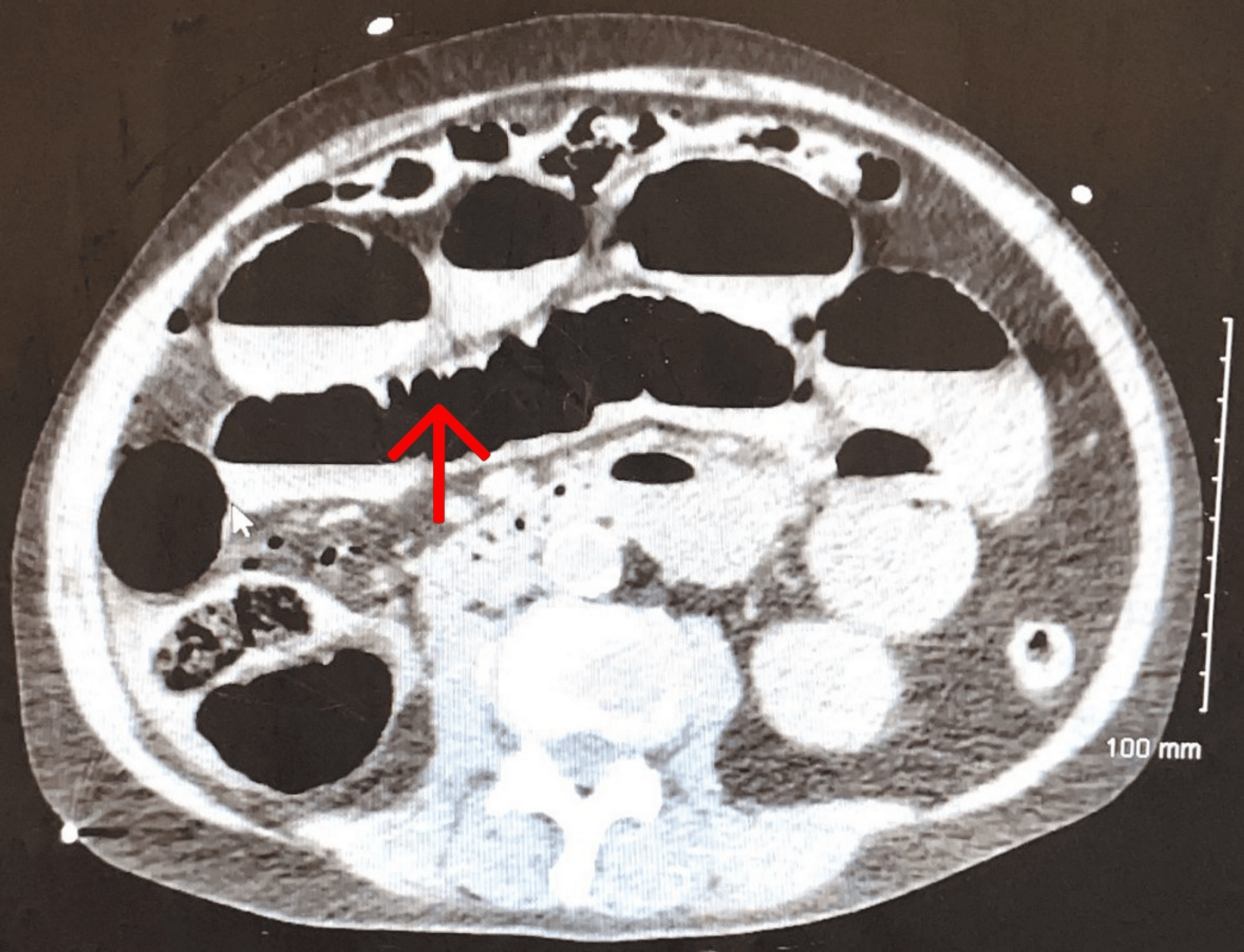
Transverse section of CT abdomen with IV contrast from emergency department showing dilated loops of small bowel.

**Figure 2:**
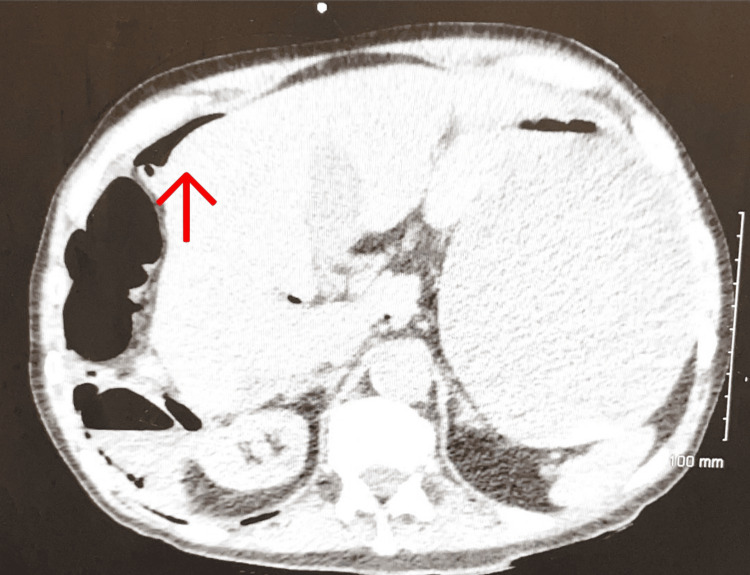
Transverse section of CT abdomen with IV contrast from emergency department showing free air in the abdomen anterior to the liver.

**Figure 3:**
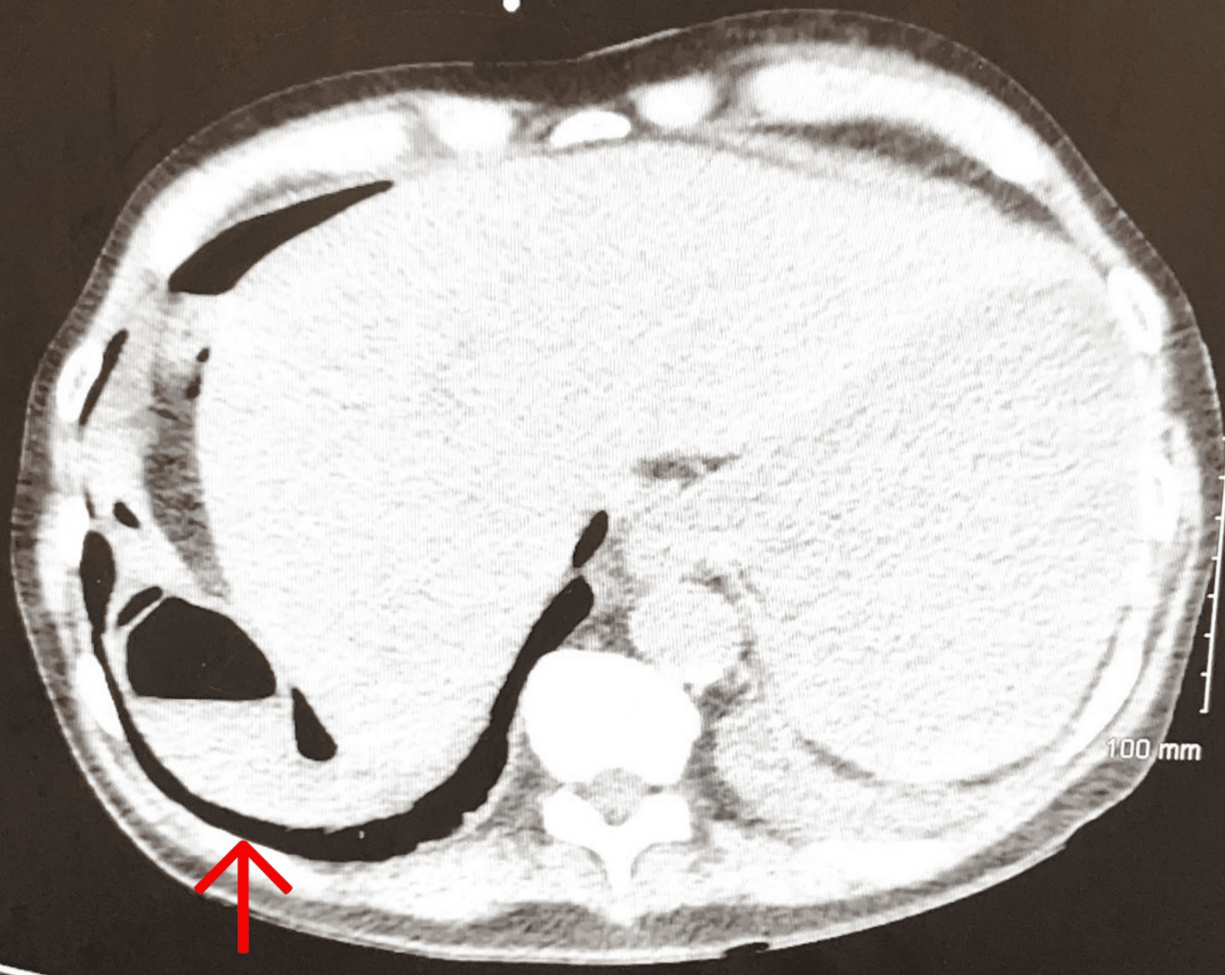
Transverse section of CT abdomen with IV contrast from emergency department showing small bowel obstruction with complex free fluid near the area of obstruction.

Pertinent lab values included lactic acid of 13.0 mmol/L and an anion gap of 27 (Table 1). CT imaging with intravenous (IV) contrast showed dilated loops of bowel (Figure 1), free intraperitoneal air anterior to the liver possibly secondary to small bowel ischemia (Figure 2), and SBO with complex free fluid near the area of obstruction (Figure 3).

**Table 1:** Pertinent laboratory values on patient presentation to the ED.

Lab	Reference range	Result
Lactic acid	0.4-2.0 mmol/L	13.0 mmol/L
Anion gap	0-16	27

The patient was diagnosed with SBO complicated by perforation, leading to septic shock secondary to perforation. Given the patient’s clinical and imaging findings, emergent laparotomy was warranted with the possibility of small bowel resection.

**Figure 4:**
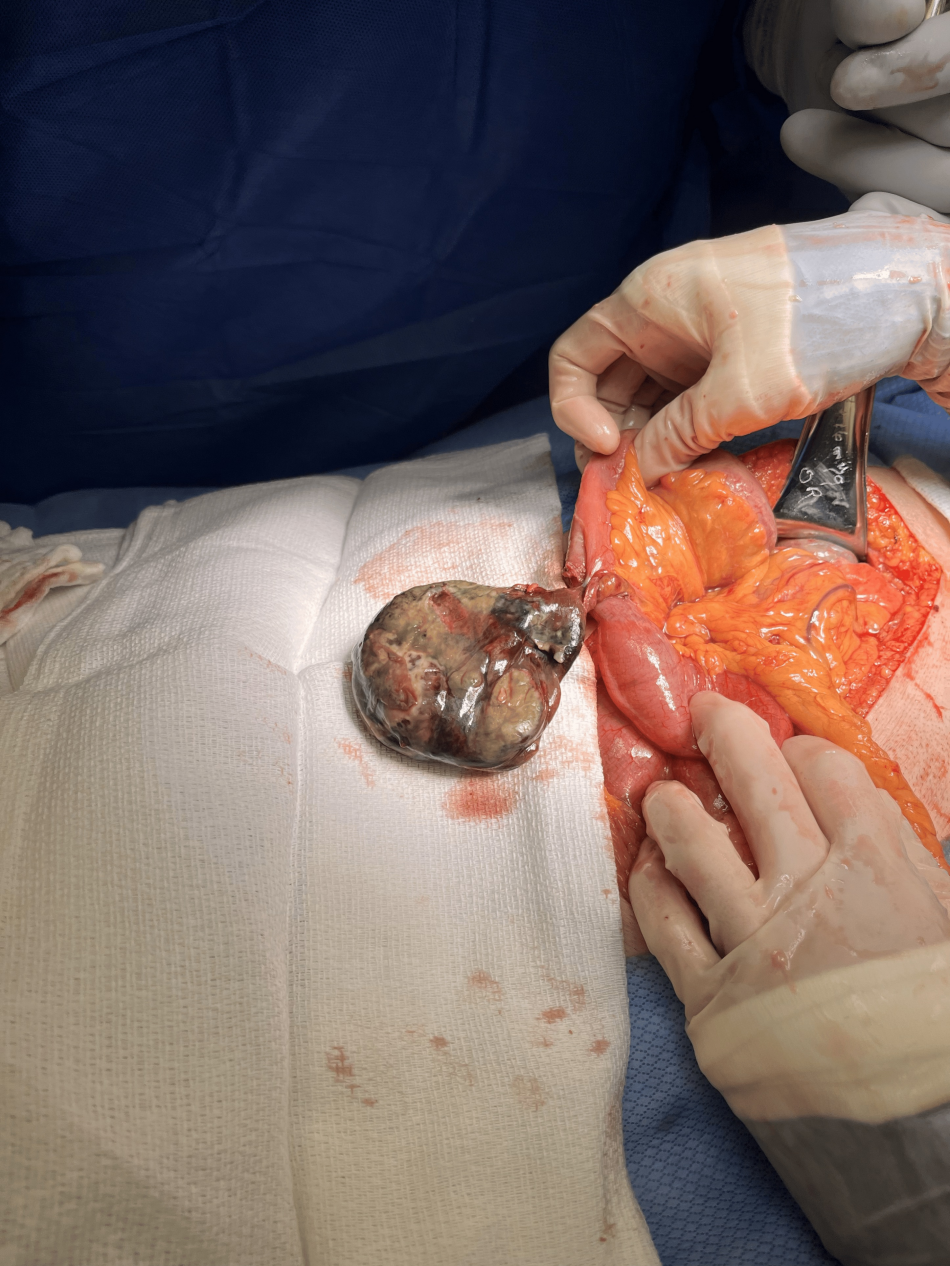
Surgical view of the small bowel showing necrosis of small bowel diverticula.

**Figure 5:**
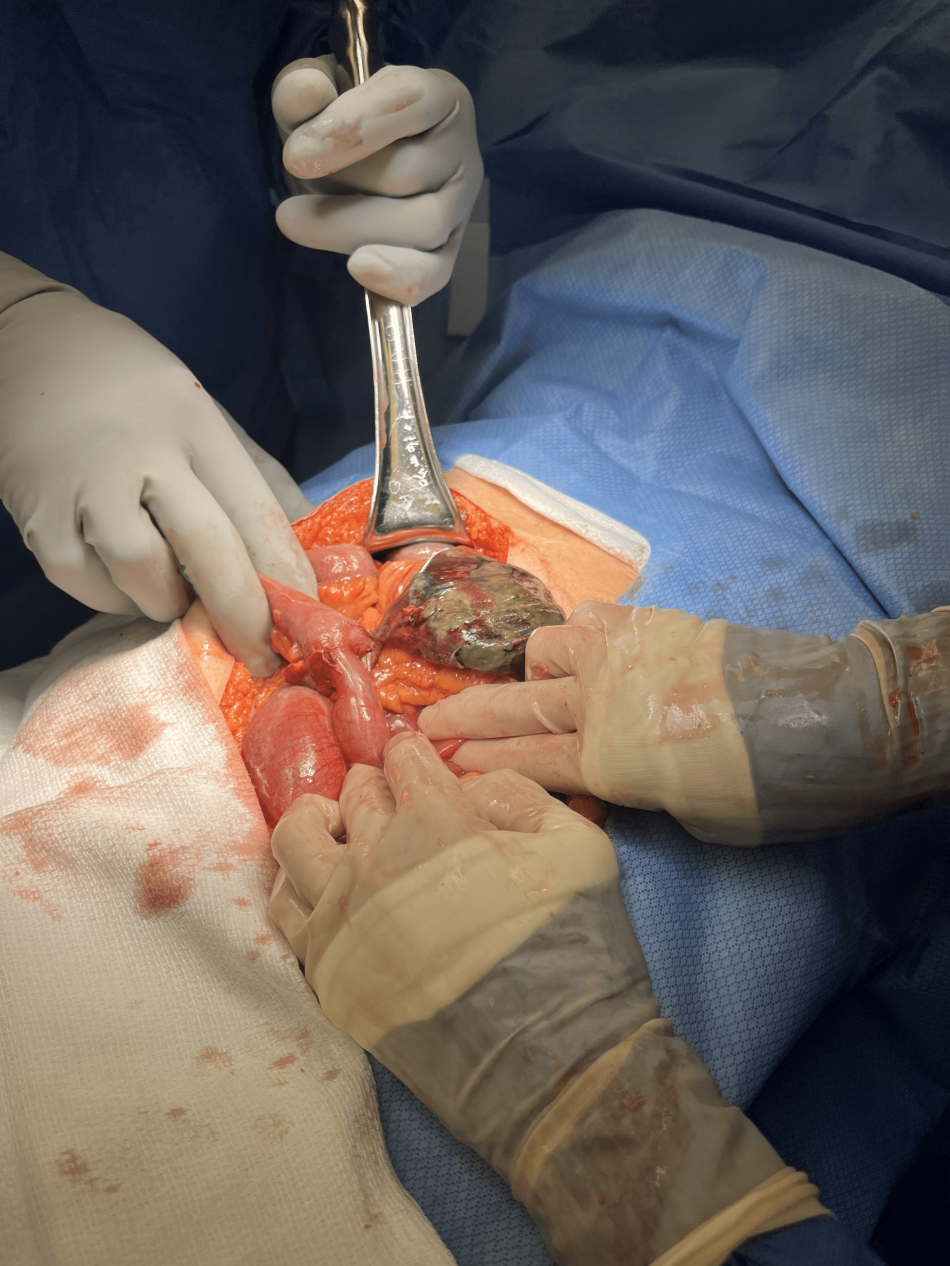
Small bowel diverticula discovered during an exploratory laparotomy.

During exploratory laparotomy, a small bowel diverticulum 1-2 feet from the ileocecal valve was discovered, firmly attached to the lateral peritoneum, liver, and another loop of small bowel. Careful finger dissection was performed and brought into the field, measuring 7.3 cm x 3.6 cm. Upon closer inspection, the diverticulum appeared partially collapsed with its serosa showing extensive necrosis and hemorrhage (Figures 4, 5). The interior of the diverticulum was filled with a large amount of hemorrhagic and necrotic debris. The diverticulum wall measured between 1 mm and 3 mm in thickness. Resection of the small bowel with primary anastomosis was ultimately performed with an excised specimen sent for pathology analysis. The post-op diagnosis was an incarcerated small bowel diverticulum with SBO.

The pathology report revealed a small bowel diverticulum with extensive transmural ischemic necrosis (gangrene) accompanied by severe acute inflammation and hemorrhage along with acute serositis in a small bowel segment. The patient was admitted to the hospital and discharged five days post-op with outpatient follow-up in the clinic. The patient had a full recovery with no additional treatment.

## Discussion

SBO is the most common diagnosis encountered by general surgeons, constituting approximately 300,000 inpatient admissions annually in the United States. Adhesions resulting from previous abdominal surgeries are the primary cause, accounting for 70% of cases. "Virgin abdomens" or those without previous surgeries typically present benignly; yet around 60% of these cases stem from congenital de novo or inflammatory adhesions. Less frequent causes of SBO include Crohn's disease, gallstone ileus, and MD [2]. While the majority of SBO cases respond to non-surgical interventions, approximately 24% necessitate surgical intervention for resolution [3]. Surgical indications encompass signs of bowel ischemia, strangulation, pneumoperitoneum, pneumatosis intestinalis, or clinical evidence of peritonitis [2]. The patient, devoid of prior abdominal surgeries and presenting with SBO, pneumoperitoneum, and sepsis including elevated lactate levels (13.0 mmol/L) and anion gap (13), required urgent laparotomy as the essential management.

During the laparotomy, a small bowel diverticulum emerged as the culprit behind the obstruction. Positioned 1-2 feet from the ileocecal valve within the ileum, it intricately adhered to the lateral peritoneum, liver, and another loop of the small bowel. Measuring 7.3 cm (2.87 in) x 3.6 cm (1.41 in), the diverticulum presented as gangrenous and necrotic, surrounded by areas covered in exudate. A subsequent small bowel resection was conducted, and the excised tissue was sent for pathological analysis to determine the root cause.

Diverticula are pouches in the mucous lining of the digestive tract, bowel, or colon [7]. Small bowel diverticula are categorized as true or false. False diverticula occurs when only the internal bowel layers protrude through the muscularis. In contrast, true diverticula involve protrusions of all three layers of the bowel [8]. While small bowel diverticula are rare and are normally asymptomatic, they can lead to a multitude of complications, such as small bowel obstruction, acute diverticulitis, or perforation, which may require surgical intervention [9]. Thus, the etiology of diverticula is essential for determining management. Despite the pathology report revealing 100% necrotic tissue, the clinical suspicion for MD remained high due to its anatomical location and other characteristic findings associated with the criteria for MD. With these criteria met, we believed MD was more likely than a false diverticula.

While MD is typically associated with children, adult cases exhibit a wide range of presentations. Symptoms may include abdominal pain, vomiting, rectal bleeding, blood in stool, fever, constipation, distension of the stomach, and fatigue. In some instances, patients may also experience hyperactive bowel sounds, tenderness to palpation in all four quadrants, and laboratory abnormalities, such as elevated liver function, C-reactive protein, and white blood cell count. Diagnostic imaging, such as CT scans, may show a fat stranding, fluid, and dilated loops of small bowel [4]. However, due to symptom overlap with other gastrointestinal conditions, such as Crohn’s disease, appendicitis, or peptic ulcers, accurate diagnosis of MD requires extensive scans or tests [10]. Plain film abdominal X-rays may be useful for detecting intestinal obstruction but are rarely of benefit when attempting to diagnose diverticula. A diagnosis of bleeding within a diverticulum can be made with Meckel's scan or Mesenteric arteriography [5].

The primary approach to managing MD involves surgical resection, although the presentation of the condition can influence the decision for surgical intervention. In cases where MD is incidentally discovered, removal is advisable under specific circumstances as follows: age over 50 years, male, diverticulum length exceeding 2 cm, presence of abnormal tissue on histopathologic examination, a broad-based diverticulum, or the existence of fibrous bands attached to the diverticulum. When MD manifests with symptoms, surgical resection is strongly recommended [5]. The decision-making process regarding surgical management hinges on various factors, emphasizing the importance of tailored approaches based on individual patient characteristics and the clinical presentation of MD.

The identification of MD on scans in the emergency scenario of small bowel obstruction and perforation presents a significant challenge for physicians. This difficulty arises from the diverticulum's variable size and location within the small intestine, intermittent manifestation of symptoms, limitations associated with common imaging modalities, symptom overlap with other gastrointestinal conditions, and the dynamic nature of small bowel obstruction. Successfully detecting MD necessitates a comprehensive approach involving a combination of imaging studies, a thorough exploration of clinical history, and a heightened clinical suspicion to ensure an accurate and timely diagnosis. The inclusion of MD in the differential diagnosis should be appreciated when evaluating patients with SBO or recurrent abdominal pain with no clear justification for the diagnosis.

## Conclusions

MD is a relatively rare condition that is commonly overlooked in adults. Consideration of MD in differentials should be included when assessing patients with unexplained SBO and recurrent idiopathic abdominal pain, especially in those without a history of abdominal surgery. A comprehensive approach to diagnosing small bowel obstruction including a wide range of potential causes, including MD, enables clinicians to make informed decisions and provide optimal patient care. Continued awareness of MD and its implications for gastrointestinal health is essential for enhancing patient management and furthering our understanding of this congenital anomaly’s impact.
